# Herpes Simplex Encephalitis of the Parietal Lobe: A Rare Presentation

**DOI:** 10.7759/cureus.785

**Published:** 2016-09-16

**Authors:** Christian Fisahn, Lara Tkachenko, Marc Moisi, Steven Rostad, Randle Umeh, Michael E Zwillman, R. Shane Tubbs, Jeni Page, David W. Newell, Johnny B Delashaw

**Affiliations:** 1 Orthopedic Surgery, Swedish Neuroscience Institute; 2 Department of Trauma Surgery, BG University Hospital Bergmannsheil, Bochum, Germany; 3 Neurosurgery, Swedish Neuroscience Institute; 4 Seattle Science Foundation; 5 Neurological Surgery, Wayne State University; 6 Pathology, CellNetix; 7 Anatomy, St. George's University School of Medicine; 8 Anesthesiology and Critical Care, Houston Methodist Hospital; 9 Neurosurgery, Seattle Science Foundation; 10 Chief of Neurosurgery, Swedish Neuroscience Institute

**Keywords:** herpes simplex encephalitis, virus, central nervous system, parietal lobe

## Abstract

A 69-year-old female with a history of breast cancer and hypertension presented with a rare case of herpes simplex encephalitis (HSE) isolated to her left parietal lobe. The patient’s first biopsy was negative for herpes simplex virus (HSV) I/II antigens, but less than two weeks later, the patient tested positive on repeat biopsy. This initial failure to detect the virus and the similarities between HSE and symptoms of intracranial hemorrhage (ICH) suggests repeat testing for HSV in the presence of ICH. Due to the frequency of patients with extra temporal HSE, a diagnosis of HSE should be more readily considered, particularly when a patient may not be improving and a concrete diagnosis has not been solidified.

## Introduction

Herpes simplex encephalitis (HSE) is a life-threatening disease caused by an infection of the herpes simplex virus (HSV) in the central nervous system. Herpes simplex type I is the most common cause of fatal sporadic encephalitis in the United States, accounting for approximately 10–20% of the 20,000 annual cases of viral encephalitis [1]. The infection occurs in all age groups. Untreated, mortality from herpes encephalitis can approach 70% and most survivors have significant neurologic deficits [1-2]. Even with appropriate diagnosis and treatment, mortality may still be as high as 20–30% [1, 3-5]. Its occurrence is not well understood, though it is thought to result either from direct infection or stress-related reactivation of the dormant virus causing subsequent aggravation of a cranial nerve with spread to the brain [6-7].

HSE most commonly presents in the temporal and frontal lobes, with extra temporal involvement seen only in advanced cases. In this rare occurrence of HSE, the patient presented with an acute parietal intraparenchymal hematoma with subarachnoid hemorrhage (SAH) originally thought to be either hypertensive or metastatic disease but ultimately determined to be HSE isolated to the parietal lobe.

## Case presentation

**Figure 1:**
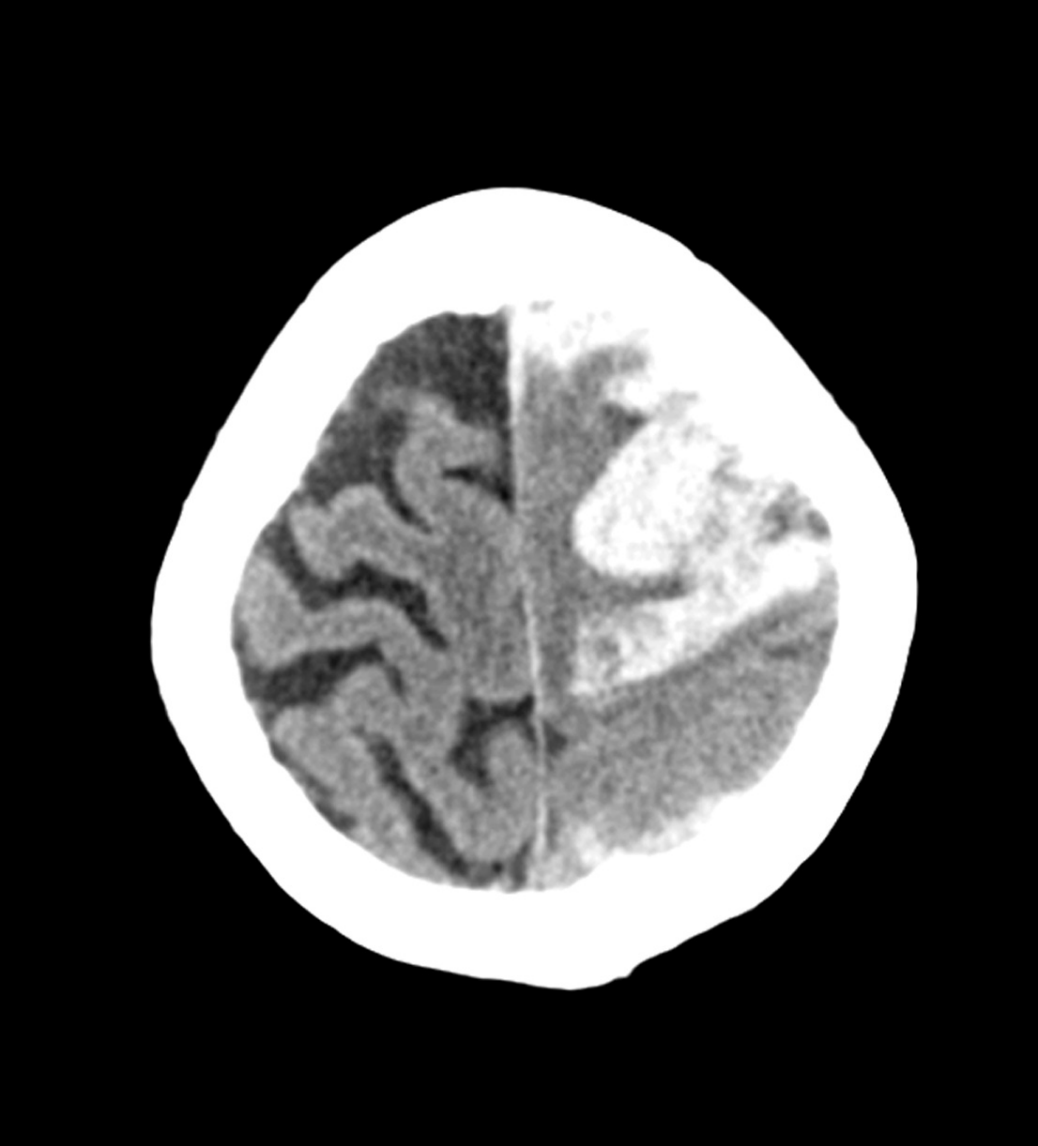
Axial non-contrast CT showing left frontoparietal hematoma

A 69-year-old female with a past medical history of breast cancer and hypertension was admitted for acute onset headache and right hemiparesis, which occurred during coitus. A computed tomography (CT) of the brain showed a large left fronto-parietal hematoma with SAH (Figure 1).

On initial examination, she was globally aphasic and hemiparetic. Subsequently, she became progressively lethargic requiring craniotomy for evacuation of the hematoma. She underwent a left frontoparietal craniotomy, which grossly demonstrated a hematoma without any clear tumor or other etiology. The pathology was consistent with a hematoma. There were no signs of metastatic disease or amyloid. The immunostains were negative for herpes simplex I/II. An angiogram was also performed yielding negative results.

**Figure 2:**
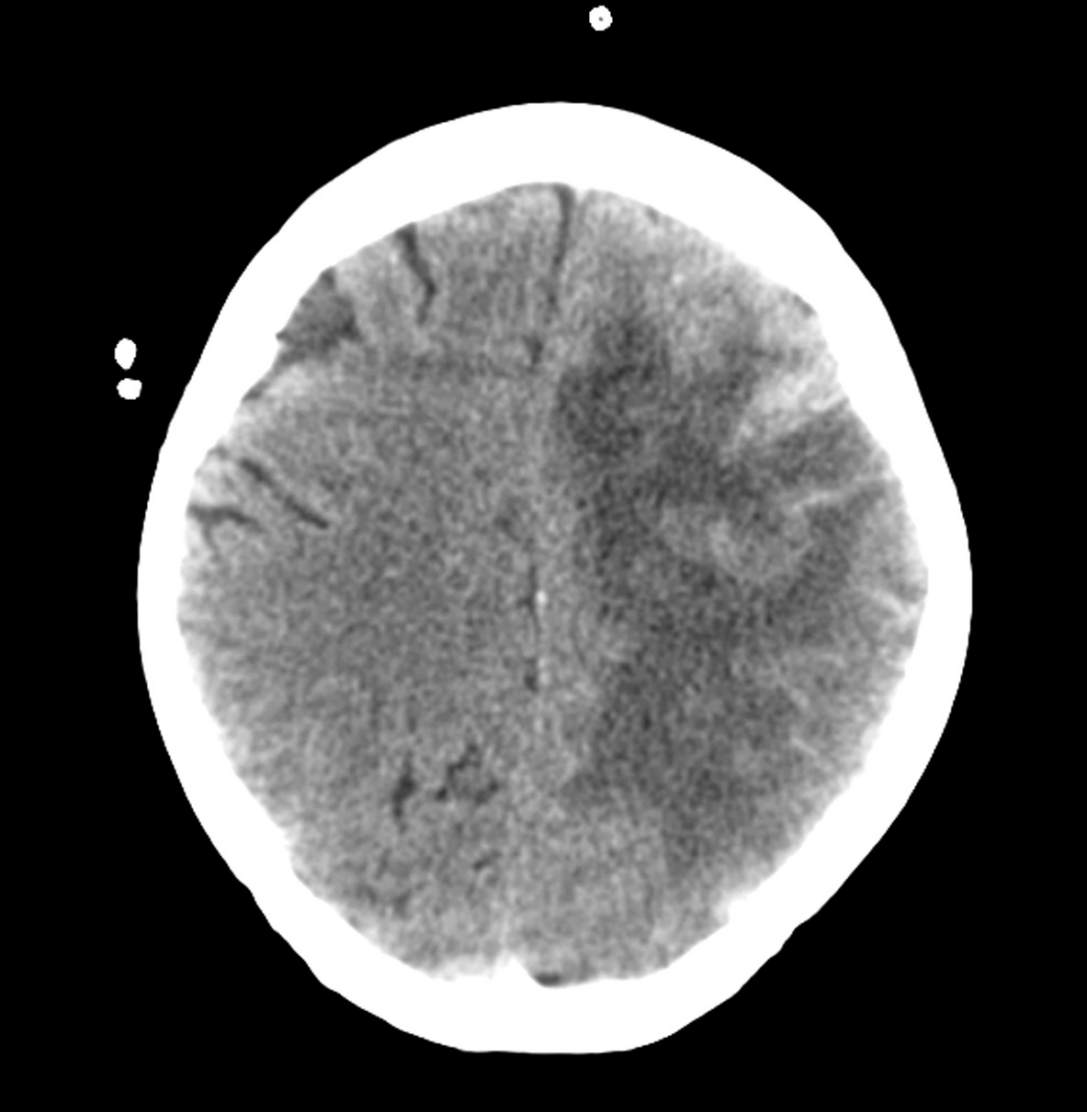
Axial non-contrast CT showing worsening cerebral edema

**Figure 3:**
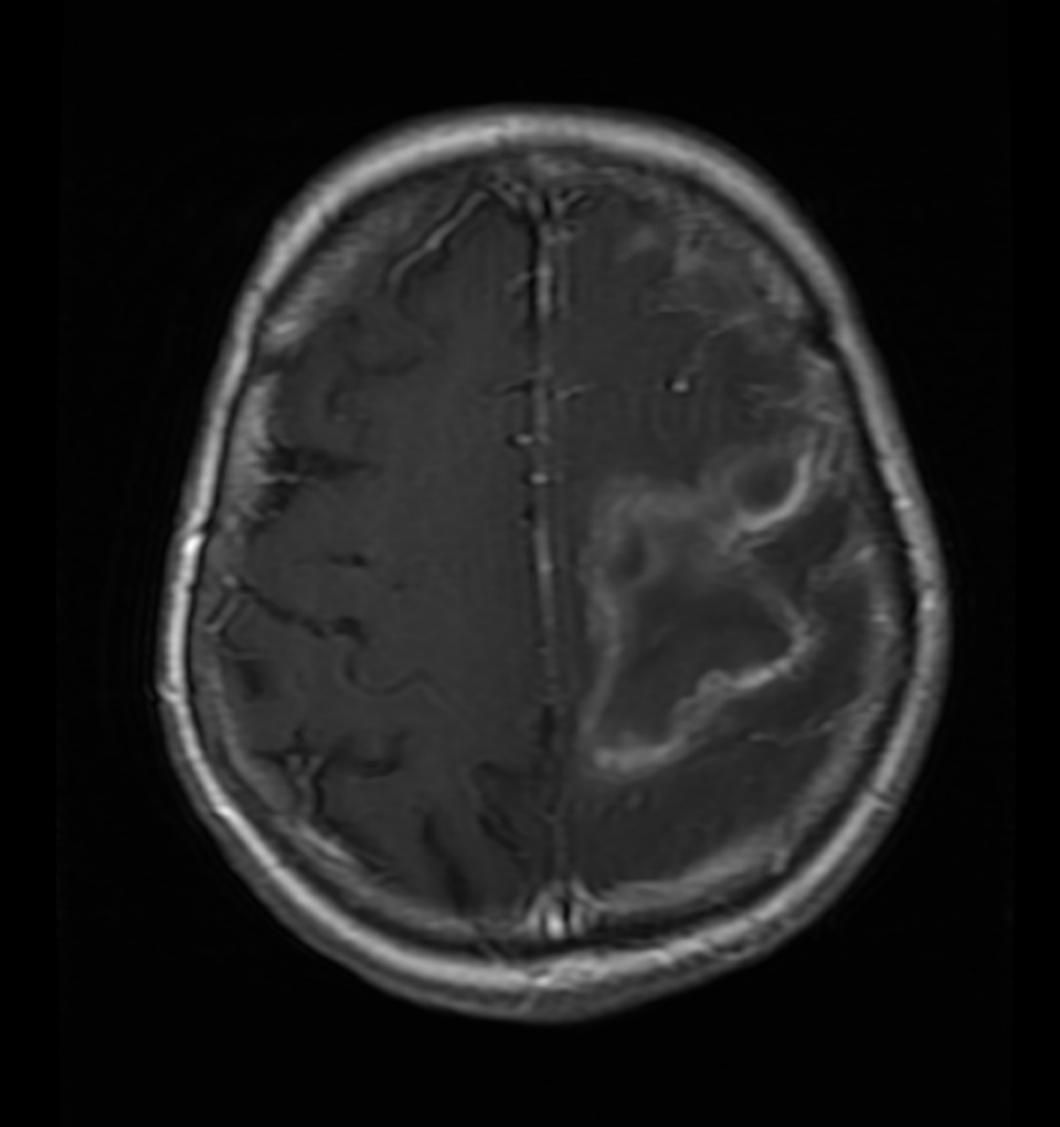
Axial postcontrast T1 weighted MRI showing worsening cerebral edema

The patient did well and was transferred to the rehabilitation unit on postoperative day five. While there, the patient became febrile and encephalopathic. The axial CT shown in Figure 2 and the postcontrast axial T1-weighted magnetic resonance imaging (MRI) shown in Figure 3 demonstrate increased cerebral edema in the region of hematoma evacuation.

She was taken back to the operating room (OR) where she underwent a left decompressive craniectomy for malignant cerebral edema and possible encephalitis. The brain tissue obtained at this time tested positive for HSV infection (see pathology). Unfortunately, subsequent postoperative imaging revealed a left frontal epidural hematoma, and the patient was returned to the OR for urgent evacuation. With her continued decline in the neurological status, the family ultimately decided to withdraw care and the patient passed away shortly thereafter. A written informed consent is not necessary for single case reports at our institution (Swedish Institutional Review Board).

### Pathology 

**Figure 4:**
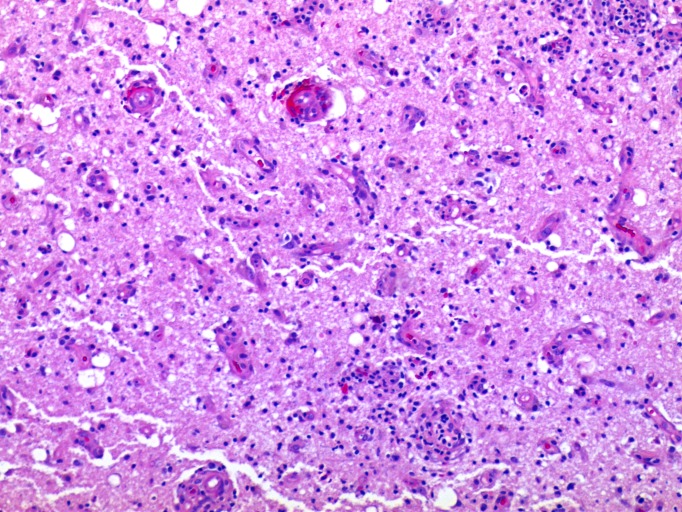
Histological examination of brain tissue A section of the cerebral cortex and white matter with perivascular and parenchymal infiltrates of lymphocytes and histiocytes. The scattered neurons show nuclear inclusions while other rare neurons show ischemic-like nuclear changes. The vessels show endothelial hypertrophy and transmural lymphocytic infiltrates (100x original magnification).

An initial brain biopsy showed acute hemorrhage consistent with intracerebral hemorrhage. A pathologic sample of cortex and white matter showed no inflammatory changes, intranuclear viral inclusions, or malignancy. The immunostains were negative for herpes simplex I/II. A second biopsy showed mixed inflammatory infiltrates within the cerebral cortex, consisting of lymphocytes, histiocytes, and neutrophils. Focal ischemic type neuronal changes were noted in the cortex. Intranuclear viral inclusions (Cowdry type A) were occasionally seen (Figure 4).

**Figure 5:**
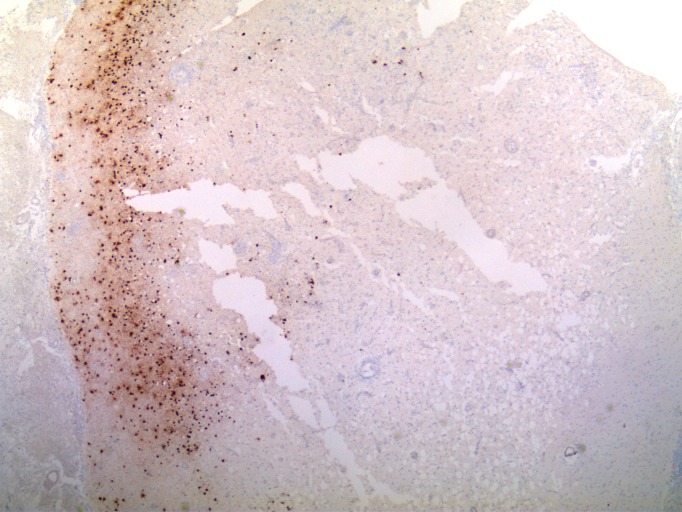
Histological examination of brain tissue Herpes simplex I/II immunostain showing regionally strong staining of infected cortical neurons. Other regions of the cerebral cortex not shown are negative for immunostaining (no infection) (40x original magnification).

**Figure 6:**
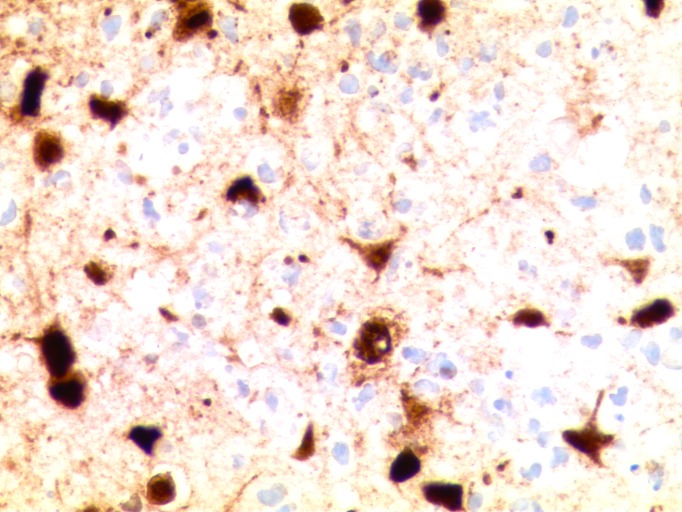
Histological examination of brain tissue High magnification of cerebral cortex with herpes simplex I/II immunostain showing strong nuclear and cytoplasmic staining of neurons as well as necrotic cells (400x original magnification).

The immunostains showed CD3-positive lymphocytes and CD163-positive histiocytes. The immunostains were positive for herpes simplex I/II infection within the cortical neurons; however, some regions of the cortex were negative for the viral infection (Figures 5-6). The immunostain to varicella zoster was negative. There was no evidence of malignancy or vasculitis.

## Discussion

The common causes of ICH include head trauma, blood vessel abnormalities (aneurysm, arteriovenous malformation, etc.), brain tumors (primary and metastatic), infection, cerebral amyloid angiopathy (CAA), and hypertension.

While there are many causes of ICH, approximately 80% of cases of ICH are considered primary ICH in which 65% are attributed to arterial hypertension and 15% to CAA, with the remaining 20% of cases being considered as secondary ICH [8]. Hypertension weakens arterial walls increasing the risk of hemorrhage. The prevalence of hypertension in patients with ICH is 46–56% [9]. In CAA, beta amyloid aggregates accumulate in the walls of cerebral vasculature increasing the risk of ICH. Hemorrhage is also characteristic of many malignancies, including melanoma, breast, lung, and choriocarcinoma metastasis, as well as glioblastomas and oligoastrocytomas (primary brain tumors). Although ICH is present in many different pathologies, it is also reported as a late stage symptom of HSE. Due to the presence of various pathologies in ICH, it is recommended that a broad and detailed differential be considered.

In one study, the clinical appearance of ICH and HSE symptoms overlapped in two-thirds of the patients [10]. In cases of HSE, hemorrhage is most commonly found unilaterally isolated to the temporal lobe, with bilateral spread occurring throughout the limbic system [11]. While more could be discussed on the relationship of hemorrhages or infarcts and viral infections (particularly varicella-zoster virus (VZV), which can cause a vascular-related inflammation and infarction), there is limited literature to comment on.

In addition to the unusual parietal location of the HSV in our patient, the occurrence of a hematoma as a complication of HSE is rare. Although it has been documented in 11 other cases, there are only two other reports in the literature in which the patient also displayed higher than normal blood pressure at admission [12]. The reasoning behind hematoma occurrence in patients with HSE is uncertain, but the suggested causation is small vessel rupture due to hypertension and vasculitis secondary to increased intracranial pressure [12]. In this particular case, the patient’s history of hypertension likely played a role in hematoma formation, and due to her acute illness, we were unaware of her HSV status.

The patient initially presented with what was thought to be a hypertensive hemorrhage or hemorrhage as a result of a breast cancer metastasis. The occurrence of ICH in a patient with a history of breast cancer leads to the assumption of brain metastasis rather than HSE. It is important to note that the differential diagnosis should be reconsidered if patients do not improve as expected. Choi, et al. [10], showed that infection was the cause of death in 23.7% of breast cancer patients, while hemorrhage was the cause of death in only 9.2% suggesting the possibility that the patient’s HSV infection may have arisen as a complication of her breast cancer. However, it is unknown whether our patient’s infection was directly related to her breast cancer or its treatments, or was simply a reactivation of the herpes virus due to stress.

The patient’s first biopsy was negative for antigens for HSV types I/II, as determined by immunohistochemical HSV types I/II staining. Testing the cortex showed acute hemorrhage with some inflammation and necrosis. The tissue displayed lymphocytic and neutrophilic inflammation, proposing the possibility of an infection or infarct. Thirteen days later, the second biopsy yielded increased inflammation and necrosis suggesting HSE with herpes simplex I/II immunohistochemical staining positive for HSE.

The quick onset and initial negative immunohistochemical staining for the HSV I/II virus are consistent with the regional involvement of the brain by infection. Similarly, the repeat biopsy of the patient led to the correct diagnosis. This raises the important issues of unusual presentations for HSV encephalitis (both as with hemorrhage and anatomic site of presentation) and the need to repeat biopsy patients who do not initially explain the clinical or radiologic picture (false negative biopsy staining for virus due to variability in the site of infection).

Typically, imaging detection of brain bleeding or abnormalities, specifically in the temporal lobe, serve as a strong indication of HSE. As seen in this patient, an HSV infection outside the temporal lobe becomes difficult to diagnose as the possibility of HSE is overlooked clinically and radiographically because of unusual features and pathologically due to sampling bias of tissue that was not infected. Therefore, this case underscores the importance of adequate sampling of radiologically abnormal tissue including brain parenchyma associated with hemorrhage or hematoma.

Despite the expectation for purely temporal lobe involvement, one small study demonstrated that 55% of patients exhibited the extratemporal presence of HSE. Furthermore, in 15% of these patients, HSE was isolated to strictly extratemporal regions [11]. Another study reported that ICH was present in the HSE involved region at admission in 35% of patients [12], suggesting that presence of HSE should be considered in the event of any ICH and despite the anticipated location of the infection. This further supports considering HSE as a possible diagnosis in the incidence of extratemporal hemorrhage. Most hematoma cases are not highly sampled by pathologists as they are mostly blood, however, if there is any brain tissue and if there is suspected underlying pathology, the tissue must be carefully examined. These different settings should lead to a broad differential requiring more extensive precautionary testing using a direct biopsy approach in contrast to a blind biopsy (without correlating radiologic abnormalities) to rule out HSE. The intraoperative frozen section procedure is an important step to confirm lesion tissue, although the diagnosis of HSE frequently will need immunostains to confirm viral presence.

## Conclusions

Due to the exceedingly high mortality rate of HSE, such a case calls for greater consideration of the presence of HSV in the occurrence of hemorrhage outside the temporal lobe. Furthermore, it suggests placing larger emphasis on the possibility of underlying HSE in the occurrence of ICH and its symptoms. Directed tissue sampling of radiographically abnormal tissue is critical to avoid false negative biopsy results as a result of sampling non-infected tissues.
